# Clinical Efficacy and Safety of Eculizumab for Treating Myasthenia Gravis

**DOI:** 10.3389/fimmu.2021.715036

**Published:** 2021-08-11

**Authors:** Hai Xiao, Ka Wu, Xiaoliu Liang, Rong Li, Keng Po Lai

**Affiliations:** ^1^Department of Neurology, Guigang City People’s Hospital, The Eighth Affiliated Hospital of Guangxi Medical University, Guigang, China; ^2^Department of Pharmacy, The Second People’s Hospital of Nanning City, The Third Affiliated Hospital of Guangxi Medical University, Nanning, China; ^3^College of Pharmacy, Guangxi Medical University, Nanning, China; ^4^Laboratory of Environmental Pollution and Integrative Omics, Guilin Medical University, Guilin, China

**Keywords:** eculizumab, generalized myasthenia gravis, mechanism, systematic review, clinical

## Abstract

Myasthenia gravis (MG) is an autoimmune disease primarily mediated by acetylcholine receptor antibodies (AChR-Ab), cellular immune dependence, and complement system involvement. Since the AChR on the postsynaptic membrane is destroyed by an immune attack, sufficient endplate potential cannot be generated, resulting in the development of a synaptic transmission disorder at the neuromuscular junction and in muscle weakness. The role of the complement system in MG has been demonstrated in animal models and clinical tests, and it has been determined that complement inhibition in patients with MG can prevent disease induction and reverse its progression. Eculizumab is a humanized monoclonal antibody that inhibits the cleavage of complement protein C5 and prevents autoimmune damage; additionally, it has received subsequent approval by the Federal Drug Administration of the United States for MG treatment. However, various concerns regarding the use of eculizumab persist. In this review, we have discussed the treatment time, cost effectiveness, long-term efficacy, and tolerability of eculizumab for MG treatment. We have also summarized historical information and have presented perspectives on this new therapeutic modality.

## Introduction

Myasthenia gravis (MG) is a chronic autoimmune disease of the neuromuscular junction (NMJ) caused by an attack of the postsynaptic membrane, primarily mediated by an acetylcholine receptor antibody (AChR-Ab) (1–4). It is characterized by recurrent and protracted dysfunction. Its symptoms are generally manifested as partial or generalized skeletal muscle fatigue. In severe cases, dysphagia, articulation, choking, and coughing may occur, causing dyspnea, and may prove to be fatal in certain cases (5, 6). The onset of MG is inextricably linked to the antibodies produced, and most MG patients harbor anti-AChR-Abs (7). The complement system plays a key role in the pathogenesis of MG. In a clinical study conducted on patients with MG, membrane attack complexes (MACs) were detected in the muscle endplate. Furthermore, in experimental autoimmune MG studies, inhibition of the complement pathway reduced MAC deposition at the NMJ, thereby reducing the damage of NMJ by AChR-Ab (8). Based on these observations, it is hypothesized that inhibition of the complement cascade in AChR-Ab patients can help prevent NMJ postsynaptic membrane damage, thereby providing a theoretical basis for the development of complement inhibitors (9).

Eculizumab is a recombinant humanized monoclonal IgG2/4K antibody that binds to human C5 complement protein and inhibits the activation of the terminal complement (10, 11). It is the world’s first targeted complement inhibitor approved for the treatment of complement-mediated diseases. It has been used in the treatment of rare diseases such as paroxysmal sleep hemoglobinuria (PNH) and atypical hemolytic uremic syndrome (aHUS) (12). Eculizumab has been approved in the European Union for the treatment of adult patients with refractory generalized myasthenia gravis (gMG) who are AChR-Ab positive. In Japan, eculizumab has also been reviewed and proposed by the Ministry of Health, Labor, and Welfare as a treatment for patients with refractory AChR-Ab-positive gMG. Additionally, in the USA and the EU, eculizumab has been used as an orphan drug for the treatment of patients with MG (13, 14). This review presents a discussion on the therapeutic efficacy and tolerability of eculizumab in patients with MG and a summary on relevant pharmacological data.

## Pathophysiology of MG

The establishment of interaction between environmental factors and genetic immune factors may be the mechanism underlying the onset of MG. Environmental factors include infection by microorganisms, such as viruses. Additionally, the use of drugs such as aminoglycoside antibiotics or d-penicillamine hydrochloride may also cause MG (15). The genetic immune factors that play a crucial role include genetic polymorphisms of different human leukocyte antigen (HLA) alleles, T cell receptors, immunoglobulins, and cytokines (16). The development and the progression of MG are associated with the activation of anti-AChR CD4+T cells and their interaction with B cells for the production of high-affinity-specific AChR-Ab. Based on different secreted cytokines, CD4+ T cells can be divided into different subtypes, in which Th1 and Th2 promote cellular immunity and humoral immunity, respectively, and Th3 participates in the establishment of immunosuppressive mechanisms (17). Specific AChR-Ab impairs the structure and function of NMJs by activating the complement system, thereby promoting AChR degradation and AChR dysfunction (18).

## Clinical Manifestation and Diagnosis of MG

The typical clinical manifestation of MG includes partial or generalized striated muscle fatigue, and muscle weakness is aggravated after exercise, necessitating the observation of rest (19–21). Symptoms fluctuate daily and are typically less severe in the morning, with severity increasing after work and in the evening. No other signs of MG in the nervous system have been reported (22, 23); thus, these symptoms are the primary basis for diagnosis. Furthermore, the detection of serum AChR-Abs is an important reference for MG. If serum tests are observed to be positive for AChR-Abs, it can be helpful in the diagnosis of MG (24). However, if tests present with negative results for AChR-Abs, MG cannot be ruled out. For example, children often have negative serum tests for AChR-Abs, even those with ocular MG. However, AChR-Ab is not the only factor that leads to the pathogenesis of MG. The presence of Titin-Ab, RyR-Ab, and MuSK-Ab is related to MG development, and changes in their expression are helpful for diagnosis (2).

## Traditional Medicine for MG

Historically, treatment options for MG have remained limited. The traditional therapeutic drugs are cholinesterase inhibitors, immunosuppressants, immunoglobulins, other non-specific immunosuppressants, and pyridine drugs (25), as listed in **Table 1**.

**Table 1:** Traditional medicine for MG.

Types	Mechanism	Representative drug
Cholinesterase inhibitors	Inhibition of cholinesterase activity, slowing down the degradation of acetylcholine, increases the amount of acetylcholine in the neuromuscular junction, increases the chance of ACh binding to AChR and prolongs binding time, improving muscle weakness.	Pyridostigmine
Pyridine	Promotes the release of Ach from the anterior membrane of neuromuscular junctions.	3-4-diaminopyridine
Antimetabolite	interferes with DNA synthesis and hampers the proliferation of leukomonocytes.	Azathioprine
Immunosuppressive agent	Inhibition of lymphocyte activation, inhibition of T cells is particularly significant, resulting in reduced AchR-ab production, resulting in the relief of most patients.	Glucocorticoid
IVIG	IVIG can replace AchRAb at the AchR site, thereby protecting AchRAb from AchR.	Immunoglobulin
Other non-specific immunosuppressants	Inhibits cellular and humoral immunity, allowing most cases to be alleviated.	CyA

### Anticholinesterase

Cholinesterase inhibitors are traditional first-line drugs used for the treatment of MG in all patients with MG, excluding those with cholinergic crisis (25). The mechanistic action of cholinesterase inhibitors involves the inhibition of the activity of cholinesterase, deceleration of the degradation of acetylcholine, augmentation in the amount of ACh in NMJs, and increase in the chances of binding of Ach to AChR, which collectively prolong the binding time and improve muscle weakness symptoms. Pyridinium bromide is a representative anticholinesterase with fewer side effects (26). The drug is only considered a symptomatic treatment and exerts no fundamental therapeutic effect on autoimmune attacks caused by NMJs. It cannot prevent the progression of the disease, leading to an epitope spread. Additionally, resistance to the drug is common and the drug should not be used for long-term treatment.

### Pyridines

Pyridines are used to promote the release of acetylcholine from the anterior membrane of NMJs. They can also improve muscle strength and neuromuscular transmission. Examples of pyridines used for MG treatment include 4-aminopyridine and 3-4-diaminopyridine (27).

### Antimetabolites

Azathioprine (AZA) is an antimetabolite that can be converted to 6-mercaptopurine by the action of thiopurine *S*-methyltransferase. AZA interferes with DNA synthesis and inhibits leukocyte proliferation. AZA is one of the most commonly used drugs considered for the treatment of MG. AZA has been used for MG treatment since 1964 (28). When AZA is used as a monotherapy, initial myasthenic reaction improvement is typically observed after 4–6 months (29). As a result, AZA monotherapy may not be a good choice for patients with serious conditions. However, combination with cortical hormones may cause rapid alleviation of symptoms. Unfortunately, deficiency in thiopurine *S*-methyltransferase can result in greater accumulation of cytotoxic 6-thioguanine nucleotides, and this further induces adverse reactions, including bone marrow suppression. A study has shown that the prevalence of the TPMT genotype is associated with adverse effects occurring due to AZA therapy in patients with MG (30). Accordingly, it is recommended that TPMT genotype or phenotype testing should be routinely performed before the use of AZA is commenced.

### Immunosuppressive Agents

Glucocorticoids are the first choice of immunosuppressive agents for patients with MG. Its primary mechanism involves the inhibition of lymphocyte activation, especially the inhibition of the proliferation of T cells, resulting in a decrease in AChR-Ab production, and enables most patients to achieve remission (4). However, this therapy is slow-acting, and there are some side effects such as osteoporosis, infection, and Cohen syndrome. The application of immunosuppressive agents can largely reduce the use of considerable doses of hormones, which may lead to the deterioration of the early stage of the disease (4).

### Immunoglobulin Therapy

Intravenous immunoglobulin therapy involves the administration of a high-dose immunoglobulin that can replace AChR-Ab at the AChR site, thereby conferring protection to AChR against AChR-Ab in patients with severe MG. It presents with few side effects and is convenient, safe, and effective (27, 28).

### Other Non-Specific Immunosuppressants

Other non-specific immunosuppressants are used to inhibit cellular and humoral immunity, such that most symptoms can be alleviated. The representative drug is cyclomycin A. However, non-specific immunosuppressants present with side effects and poor efficacy (29).

## The Complement System in MG

The lack of availability of effective treatments and a better understanding of the pathophysiological mechanisms of the disease motivates drug developers to explore new therapeutic drugs for MG. Ozawa et al. (31) analyzed the changes in serum complement levels and their regulators in patients with MG. They found that patients with MG harbored higher soluble C5b-9 and vitronectin levels than patients in the control group. However, serum properdin levels were lower than those in the control group. After treatment, the indices returned to their original baseline levels. Interestingly, C5a levels were positively correlated with MG severity according to the activities of daily living (ADL) scale (32). Components of the complement system can be considered as markers for diagnosis and disease, as well as targets for therapy. The complement system, an important component of innate immunity, consists of three distinct pathways, including the classical pathway (CP), lectin pathway (LP), and alternative pathway (AP), and all converge at the common central complement component C3. CP and LP are triggered by antibodies released due to exposure to pathogens (such as gMG) and mannose-containing sugars, respectively. AP is unique because spontaneous self-activation always occurs and can be further triggered by bacterial components such as lipopolysaccharides and bacterial toxins (33). Activation of each pathway results in the formation of a C3 convertase (C3bBb) that catalyzes the cleavage of C3, resulting in chemotaxis and opsonization with C3a and C3b, respectively. The production of a considerable levels of C3b leads to the formation of C5 convertase (C3bBbC3b). In turn, C5 convertase catalyzes the cleavage of C5, resulting in the formation of anaphylatoxins, C5a and C5b, and these bind to complement proteins C6, C7, C8, and C9 to enable the formation of MAC or C5b-C9 products (C5b-C9), subsequently leading to cell lysis. As mentioned above, the formation of MAC from C3b, C5b, and other terminal complement proteins is a common concluding point for CP, LP, and AP (34). Therefore, regardless of the stimuli, blockade of C5 to prevent the conversion of C5 to C5a and C5b will effectively terminate the complement cascade. Additionally, this pathway is located downstream and avoids the impairment of C3b-mediated opsonization, immune protection, and immune regulation of immune complex clearance (35, 36). Once C5 was identified as the optimal target, a murine anti-human C5 monoclonal antibody panel was established and screened for its ability to inhibit complement-mediated lysis and to effectively block C5a production (37, 38). Monoclonal antibodies were detected in these panels. The complementarity-determining region was cloned and transplanted into human heavy and light chain antibody frameworks to generate humanized monoclonal antibodies.

## The Development of Eculizumab

**Figure 1:**
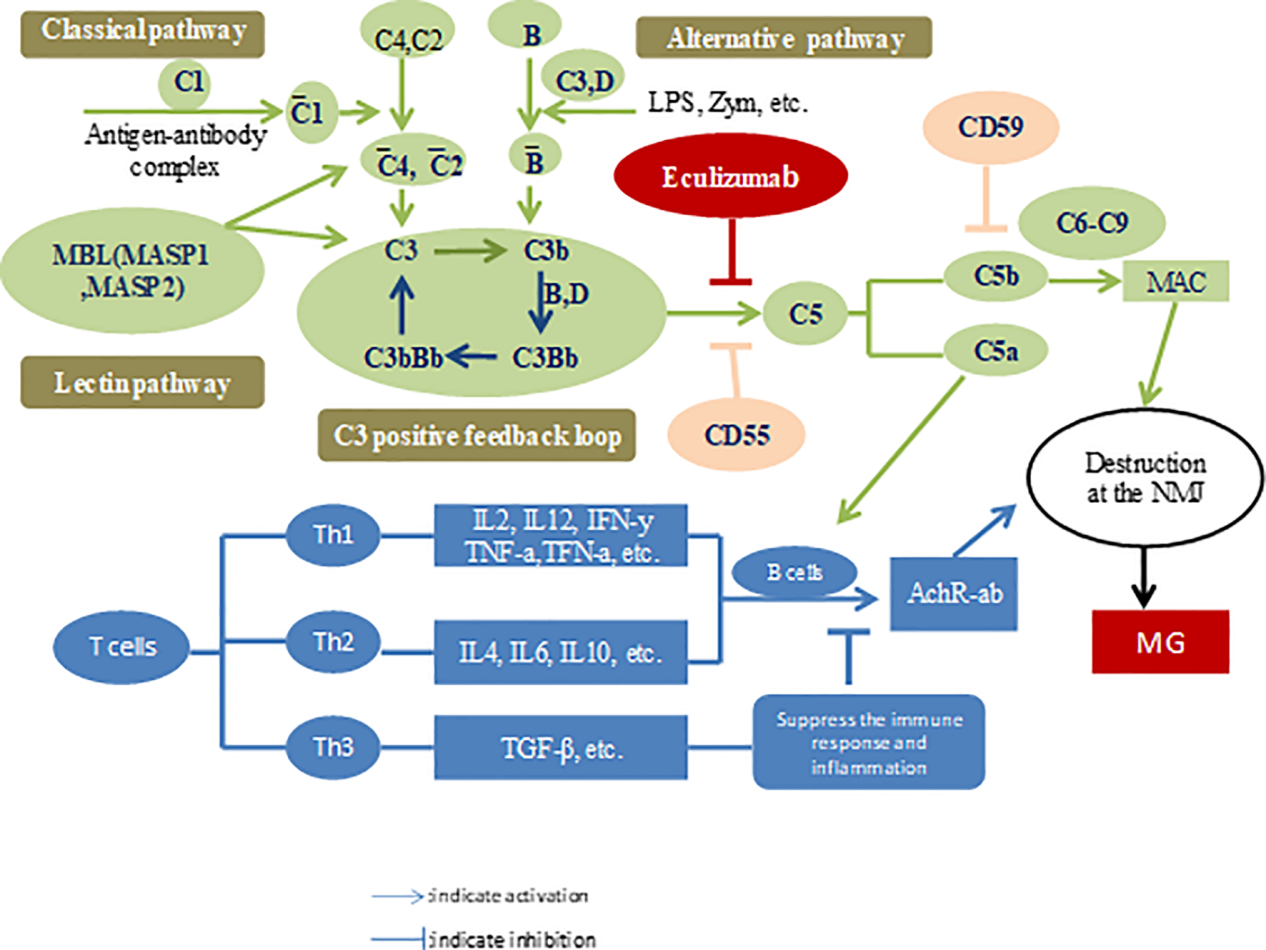
Pharmaceutical effects of eculizumab for treating myasthenia gravis.

Eculizumab has been shown to exert an *in vivo* effect in a murine model and demonstrates safety in individuals with rheumatoid arthritis, systemic lupus erythematosus, or coronary artery disease (39, 40). Antibodies are known to activate the CP, ultimately resulting in the deposition of C5b-C9 on the postsynaptic membrane, thereby destroying the AChR. Owing to chemotaxis and cell lysis associated with complement activation, the postsynaptic membrane presents with reduced sensitivity to the released acetylcholine, which inhibits nerve impulses. Therefore, eculizumab, a C5 complement inhibitor, has gained prominence as an emerging therapeutic agent for MG (41) (**Figure 1**).

### Clinical Trials of Eculizumab in Patients With MG

**Figure 2:**
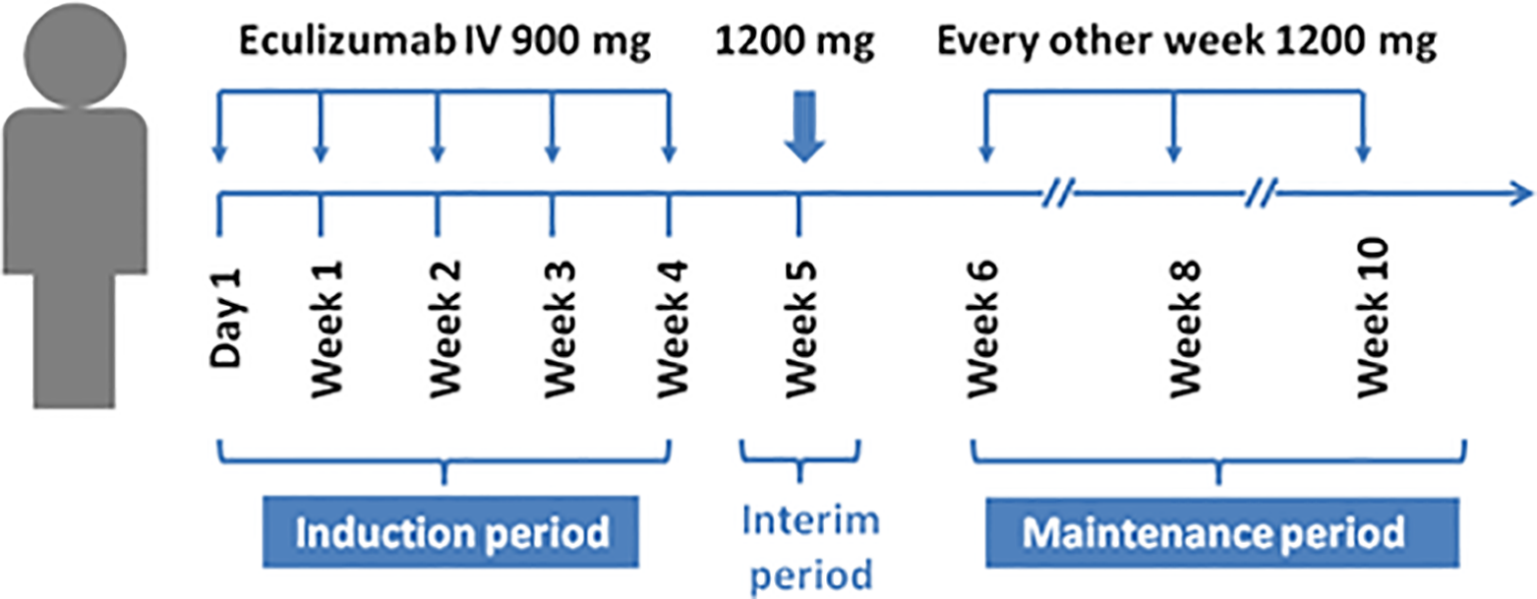
Clinical trials of eculizumab in patients with myasthenia gravis.

Results obtained from a phase 2 study have suggested that eculizumab is a terminal complement inhibitor that generates and demonstrates clinically significant improvements in patients with anti-AChR-Ab-positive refractory gMS (18) (**Table 2**). This study was conducted at 76 medical centers in 17 countries (41). The inclusion criteria were adults, an MG-ADL score of 6 or more, American MG association (MGFA) II-IV, inoculation with *Neisseria meningitidis*, receipt of at least two types of immunosuppressive therapy or immunosuppressive therapy plus chronic intravenous immunoglobulin or plasma exchange, and the lack of participation in an effective symptom control for 12 months. Patients were randomized to receive intravenous eculizumab or placebo for 26 weeks (41). The dose of eculizumab was initially 900 mg, which was gradually increased to and remained at 1200 mg after 4 weeks (**Figure 2**) (41). The primary endpoint of the study was the change in MG-ADL scores. The study enrolled 125 patients, including 62 in the treatment group and 63 in the control group. No significant difference was observed between eculizumab and placebo recipients for the primary endpoint according to the pre-specified primary endpoint analysis (least squares mean rank 56.6 [SEM=4.5] vs. 68.3 [SEM=4.5]; rank-based treatment difference=−11.7, p = 0.0698) (41). No cases of meningococcal infection or death were observed during the study. The study concluded that eculizumab did not improve the MG-ADL scores in patients with refractory systemic MG who were AChR-Ab-positive, but prespecified and *post hoc* sensitivity analyses indicated that eculizumab was effective in improving symptoms of disease, and its treatment was well tolerated and could partially alleviate the disease progression of patients (41).

**Table 2:** Summary of the clinical trials on eculizumab in myasthenia gravis.

Phase	Years	Clinical trial	Duration	Patients	Treatment	Outcomes
Phase II	2013 (18)	Multicenter, randomized, double-blind, placebo-controlled, crossover	16 weeks	14	IV 600 mg weekly for 4 wk then every other week 900 mg	At the end of period 1:
				eculizumab/placebo=7		QMG(-3 point):eculizumab-treated =86% (6/7)
				placebo/eculizumab=7		placebo-treated=57% (4/7)
						MG-ADL (-2 point):eculizumab-treated 86% (6/7)
						placebo-treated =57% (4/7)
Phase III	2017 (41)	Multicenter, randomized, double-blind, placebo-controlled, REGAIN study	26 weeks	125	IV 900 mg weekly for 4 wk then every other week 1200 mg	Prespecified worst-rank ANCOVA score:
				eculizumab=62		MG-ADL: eculizumab vs placebo=56.6 vs 68.3, *P*=0.0698
				placebo=63		QMG: eculizumab vs placebo =54.7 vs 70.7, *P*=0.0129
						MGC: eculizumab vs placebo =57.3 vs 67.7, *P*=0.1026
						MG-QOL15: eculizumab vs placebo =55.5 vs 69.7, *P*=0.0281
						The change of Neuro-QOL Fatigue total score from baseline to 26 weeks (42): eculizumab vs placebo =-16.3 vs -7.7
Phase III Extension	2019 (43)	The Open-label extension trial of REGAIN study	4 years	117	Every other week 1200 mg	The change of mean total score from baseline to open-label:
				eculizumab/eculizumab=56		eculizumab/eculizumab: MG-ADL: *P*=0.0990; QMG: *P* =0.8949; MGC:
				placebo/eculizumab =61		*P* =0.1531; MG-QOL15: *P* =0.4756
						placebo/eculizumab: all the rating scale adopted was improvement, all *P*< 0.0001
						The change of Neuro-QOL Fatigue total score from baseline to 52 weeks (42): eculizumab vs placebo =-17.5 vs -15.7

IV, intravenous; QMG, quantitative myasthenia gravis scale; MG-ADL, myasthenia gravis activities of daily living scale; MGC, myasthenia gravis composite scale; MG-QOL15, myasthenia gravis quality of life questionnaire; Neuro-QOL, Fatigue quality of life in neurological disorders.

Notably, two recently published retrospective analyses closely simulated clinical practice. James et al. reported the analysis of 98 patients with gMG with a mean follow-up period of 2.7 years (42). All patients were subjected to eculizumab therapy and had undergone prior therapy (cholinesterase inhibitors, oral corticosteroids, or other immunosuppressive treatments) at the initiation of treatment. The participants were divided into an eculizumab group and a control group. Remission was achieved in most patients, and the disease was controlled to a certain extent in each patient by week 12 of eculizumab treatment, according to the MG-ADL and quantitative myasthenia gravis (QMG) scores (42). Renato et al. analyzed patients with refractory anti-AChR-Ab-positive gMG (44). Patients experienced rapid improvements in total scores and in all four domain scores of both the MG-ADL and QMG scales with eculizumab treatment. These therapeutic effects were sustained for 2 years (44). Both publications highlighted that eculizumab treatment elicited rapid and sustained improvements in muscle strength and quality of life in patients with MG. As time has elapsed, there is insufficient information to formulate specific recommendations about the discontinuation of eculizumab tapering, and the clinical efficacy of eculizumab will continue to be evaluated. The starting dose of eculizumab is generally 900 mg on day 1 and at weeks 1, 2, and 3. The dose can be increased by 1200 mg in 4 weeks, and can be provided as maintenance dosing. The overall prevalence of headache and nasopharyngitis was 44% and 38.5%, respectively, in a study involving 117 patients with MG (43). Other common side effects include diarrhea, upper respiratory infection, and arthralgia. Notably, no cases of meningococcal infection, one of the most serious complications, were reported in the above-mentioned studies. Subsequently, the open-label extension (OLE) study of phase 3 REGAIN was designed to evaluate the long-term safety and efficacy of eculizumab in patients with gMG. A total of 92.9% of REGAIN patients first underwent a 4-week blinded induction phase, and the OLE study was commenced at week 5, extending to a period of 4 years. During the interim analysis period, the majority of patients (75%) achieved GM exacerbation remission, and improvement was observed in all rating scales adopted (43). However, eculizumab should not be discontinued abruptly without tapering, as marked MG relapses may occur (45). In one case report, a man with MG successfully exhibited responses to eculizumab, but relapses and acute severe worsening of myasthenic symptoms were observed 2 months after discontinuation. This is a critical reminder for doctors to focus on MG relapse after eculizumab withdrawal.

### Side Effects and Safety of Eculizumab

Eculizumab treatment elicits rapid and sustained improvements in muscle strength across ocular, bulbar, respiratory, and limb/gross motor muscle groups, and in associated daily activities in patients with refractory AChR-Ab-positive gMG. Eculizumab was successfully used for the treatment of gMG disease that was refractory to intravenous immunoglobulin (IVIG), plasmapheresis, myasthenia crisis, and thymoma-associated disease (46, 47). Researchers in Japan considered differences in the MG based on age, clinical characteristics, and human leukocyte antigen alleles between Asian and Caucasian patients. They investigated the efficacy and safety of eculizumab in Japanese and Caucasian participants. Subgroup analysis showed that the efficacy and the safety of eculizumab were similar in both races (48). Thus, race may not be a factor affecting eculizumab efficacy. Additionally, eculizumab seemed to present with a favorable benefit-risk profile in pregnant patients and no treatment-related adverse reactions in either the patient or the infant. Furthermore, the patients remained neurologically stable after 5 years of treatment (49). Notably, these studies recruited a low number of patients and the findings should be considered with caution. Therefore, further clinical trials are necessary.

The common side effects of eculizumab include headache and pharyngitis, followed by upper respiratory tract infection and nausea, and all were mild to moderate in severity (50). In general, eculizumab was well tolerated, and a change in the eculizumab treatment schedule due to the occurrence of side effects was not necessary. If the patient experiences mild symptoms, they are generally subjected to treatment with symptomatic drugs such as acetaminophen or paracetamol (51). Overall, patients discontinued treatment owing to the development of serious side effects such as MG exacerbation and myasthenic crisis. Plasma-exchange therapy can be implemented in MG crisis. The primary cause of death in patients with GM is the development of refractory complications. Murai et al. provided a real-world case in which two patients succumbed to the disease during the follow-up period (52). One cause of death was attributed to asphyxia during an MG crisis. The second patient who succumbed to ventricular fibrillation and acute myocardial infarction patients had a history of type 2 diabetes and hypertension (52). The death and causal relationships with eculizumab are unclear. Meningococcal infection is regarded as the most serious potential complication because the inhibition of C5 impairs opsonophagocytic activity. Eculizumab should not be discontinued without tapering, as fatal MG relapses may occur.

## The Future of Complement Inhibitors

The discovery of eculizumab and its beneficial effects observed in the treatment of MG motivated researchers to develop anti-complement drugs, resulting in the development of new agents targeting the complement system. Zilucoplan is considered an effective C5 complement pathway blocker that can specifically be used to prevent downstream complement action through the following dual mechanism: 1) inhibition of the cleavage of C5 by C5 convertase into more C5 complements, and 2) facilitation of the binding with the domain C5, thereby enforcing a blockade on the binding of C5b with the complement component C6 (53). Patients can self-administer zilucoplan, and an injection volume of less than 1 mL is required (54). Verification of the clinical effect of zilucoplan in MG will be further assessed through the open-label long-term extension of the phase 2 study and a forthcoming phase 3 clinical program. Wioleta et al. (55) screened anti-C5 mAbs from complement-deficient mice. Three such C5-blocking mAbs, such as eculizumab, are deemed efficient inhibitors of the complement cascade but differ from eculizumab, a molecule which has binding sites and other properties. The cross-reactivity of these mAbs (4G2, 7D4) highlights them as powerful tools for the conduction of proof-of-concept studies. All three antibodies are strong candidates for consideration as therapeutic tools. Linda et al. described the development of a subcutaneously administered N-acetylgalactosamine (GalNAc)-conjugated small interfering RNA (siRNA) targeting the C5 component of complement that could silence C5 expression in the liver (ALN-CC5) (56). Additionally, siRNA therapeutics are potential therapeutic agents for the treatment of MG and other complement-mediated disorders.

## Potential Alternative Complement-Based Treatment Methods

Cost and convenience are the limiting factors in the expansion of the use of anti-complement agents. Additionally, the treatment process is challenged by various factors, such as poor patient compliance and intravenous injection. Another limiting factor is that the underlying generation of autoantibodies is altered by the therapy and, therefore, discontinuation of inhibitor therapy would be expected to lead to the rapid return of myasthenic symptoms. The involvement of the complement system in the pathogenesis of MG depends on the IgG subtype. MuSK is primarily derived from the IgG4 subtype that does not activate the complement system; hence, complement inhibitors do not exhibit reactions with MuSK antibody-positive MG (57). Defects in complement therapy have motivated the development of novel drugs to be used in the treatment of MG. B-cell-activating factors may also be considered a potential therapeutic target for MG. Belimumab is an immunoglobulin antibody that binds and blocks the B-cell activating factor, leading to a reduction in B-cell differentiation *via* direct reduction in the number of B cells to alleviate dysimmunity (58). Iscalimab is an anti-CD40 monoclonal antibody that does not reduce the proportion of B cells but triggers a blockade of T cell-dependent antibody responses. Notably, a double-blind, placebo-controlled, phase II study of gMG and MuSK antibodies demonstrated high levels of safety (59). Pharmacological studies have shown that the principal mechanisms of these drugs include the reduction of leukocytes or interference with other pathways but not the blockade of the complement system. Presently, an increasing number of drugs are expected to be added to the list of drugs to be used for treatment as research is ongoing and results are awaited.

## Conclusion

Recent studies conducted on MG have focused on the identification of new targets and the development of new therapies, especially for patients with MG who are unresponsive to conventional therapies. These patients do not exhibit improved MG symptoms or significant side effects after subjection to treatment with corticosteroids and at least two other immunosuppressive agents. Patients with refractory MG continue to demonstrate symptoms that adversely affect MG-ADLs and may experience frequent episodes that may be life-threatening. Eculizumab is deemed a novel therapy and is the first adult gMG (USA), refractory gMG (EU), gMG high-dose IVIg, or PLXL, posing challenges for the use of targeted complement inhibitors (Japan) approved for anti-AChR-Ab positivity. Although MG guidelines were updated prior to the approval of eculizumab, the latest guidelines issued by the German Neuropathology Association include eculizumab as a treatment for patients with severe, refractory gMG. Currently, there are insufficient long-term data for the assessment of safety and efficacy. However, existing and recent evidences suggest that eculizumab may be a valuable emerging therapy for patients with refractory gMG.

## Author Contributions

RL and KL for research project with conception, organization, and execution. HX, KW, and XL for statistical analysis with design, execution, review and critique. RL and KL for manuscript preparation with writing of the first draft, review and critique. All authors contributed to the article and approved the submitted version.

## Conflict of Interest

The authors declare that the research was conducted in the absence of any commercial or financial relationships that could be construed as a potential conflict of interest.

## Publisher’s Note

All claims expressed in this article are solely those of the authors and do not necessarily represent those of their affiliated organizations, or those of the publisher, the editors and the reviewers. Any product that may be evaluated in this article, or claim that may be made by its manufacturer, is not guaranteed or endorsed by the publisher.

## Abbreviations

MG, myasthenia gravis; AChR-Ab, acetylcholine receptor antibodies; NMJ, neuromuscular junction; AChR, acetylcholine receptor; ANCOVA, analysis of covariance; ADL, activities of daily living; HR-QOL, health-related quality of life; MuSK-Ab, muscle-specific tyrosine kinase antibody; LRP4-Ab, low-density lipoprotein receptor-related protein 4 antibody; Titin-Ab, anti-building protein antibody; MAC, membrane attack complexes; PNH, paroxysmal sleep hemoglobin; aHUS, atypical hemolytic uremic syndrome; HLA, leukocyte antigen; CP, classical pathway; LP, lectin pathway; AP, alternative pathway; MGFA, myasthenia gravis association; QMG, quantitative myasthenia gravis; siRNA, small interfering RNA.
